# In vitro anti-plasmodial activity of three selected medicinal plants that are used in local traditional medicine in Amhara region of Ethiopia

**DOI:** 10.1186/s40360-023-00672-z

**Published:** 2023-05-11

**Authors:** Yenesew Wudu Ejigu, Bedilu Linger Endalifer

**Affiliations:** 1 Department of Pharmacy, College of Medicine and Health Sciences, Wollo University, P.O.Box: 1145, Dessie, Ethiopia; 2 Department of Pharmacy, College of Medicine and Health Sciences, Wollo University, Dessie, Ethiopia

**Keywords:** Aloe weloensis, Lepidium sativum, Lobelia gibberoa, 80% methanol, Plasmodium falciparum, Latex, In vitro, Anti-plasmodial

## Abstract

**Background:**

The plants *Aloe weloensis, Lepidium sativum*, and *Lobelia gibberoa* have been used in Ethiopian folklore medicine to treat various diseases including malaria.

**Method:**

The in vitro anti-plasmodial activity of the three crude extracts was evaluated using parasite lactate dehydrogenase assay against the chloroquine (CQ)-sensitive D10 and the chloroquine (CQ)-resistant W2 strains.

**Result:**

The methanolic extract of *L. gibberoa* roots showed the highest in vitro anti-plasmodial effect against both D10 and W2 *Plasmodium falciparum* strains with IC_50_ value of 103.83 ± 26.17 µg/mL and 47.11 ± 12.46 µg/mL, respectively. However, the methanolic extract of *L. sativum* seeds and the leaf latex of *A. weloensis* were not active with an IC_50_ value > 200 µg/mL against both D10 and W2 strains.

**Conclusion:**

The methanolic extract of *L. gibberoa* roots showed a promising in vitro anti-plasmodial activity against the CQ-sensitive (D10) and CQ-resistant (W2) strains of *P. falciparum*. Thus, the anti-plasmodial activity of this plant partly justifies and may also support the traditional use against malaria. However, the methanolic extract of *L. sativum* seeds and the leaf latex of *A. weloensis* did not exert suppressive activity on the growth of *P. falciparum* strains.

## Background

Malaria is one of the oldest recorded and major devastating and lethal parasitic diseases in the world [1]. It is a protozoal disease caused by parasites of the genus *Plasmodium* and transferred to humans by bites of certain species of infected female Anopheles mosquito [2]. *Plasmodium falciparum*, *P. vivax*, *P. ovale* and *P. malariae* are the four human *Plasmodium* species transmitted from person to person. Recently, zoonotic transmissions with the monkey malaria parasites *P. knowlesi* are increasingly being reported from the forested regions of South-East Asia, particularly the island of Borneo [3]. *P. falciparum* and *P. vivax* malaria cause the greatest public health challenge [4]. From the five of human *Plasmodium* parasites, *P. vivax* and *P. ovale* form dormant stages in the liver (hypnozoites) that can cause a clinical relapse many months after the first event [5]. According to the World Health organization (WHO) latest estimates of 2022, about 247 million cases in 84 malaria endemic countries and 619 000 deaths of malaria occurred globally. The largest cases occurred in the African region (95%), followed by the South-East Asia region (2%), and the Eastern Mediterranean region (2%). The African region still carries an excessively high share of the global malaria burden 2022 [6]. Malaria remains an important cause of illness and death in children as well as adults (3). However, children under 5 are particularly vulnerable to infection, sickness, and death; more than two thirds (67%) of all malaria deaths occur in this age group [6]. It is still one of the leading causes of morbidity and mortality in some of the poorest tropical and subtropical regions. Particularly, it remains to be one of the most significant illnesses in sub-Saharan Africa, where 20% of children < 5 years old die as a result of this infection [7, 8]. Therefore, malaria control needs an integrated approach including prevention (mainly vector control) and prompt treatment with effective antimalarial agents [3]. Besides, malaria also causes a great economic impact with a total fund of an estimated US $ of 2.7 billion in 2018 for malaria control and elimination [6].

Nowadays, there is a problem of drug resistance of malaria parasites even to artemisinin, which is the main ingredient in most effective malaria treatment; Artemisinin-based combination therapy (ACT), puts at risk the gains that we have made to date to combat malaria, and may seriously jeopardize further progress in malaria control and elimination [9]. Besides, despite some promising efforts of malaria control through a vaccine, the success rate is still limited [7, 10]. Thus, the world is in urgent need of new anti-malarial drugs [11].

In discovering new anti-malarial drugs, history has taught us how the knowledge of traditional medicines is valuable [12–14]. Despite widespread development of resistance and difficulties in poor areas to afford as well as to access effective anti-malarial drugs, currently used and potent anti-malarial drugs such as artemisinin and quinine are obtained from plant sources. Hence, it is imperative to focus on traditionally used medicinal plants for the discovery of new anti-malarial sources for the future [8, 12].

It is predicted that over 1,200 plants worldwide are reported to possess anti-malarial activities [15]. The present experimental medicinal plants have a traditional claim for the treatment of malaria. In various countries of the world, different parts of *L. sativum* are believed to be an effective medicinal remedy. The seeds are used in treating dysentery and diarrhea [16], migraine [17], asthma, bronchitis cough [18], hypertension [19], rheumatic arthritis, muscular pain, bacterial and fungal infections, blood and skin disease, and tumors [20]. The seeds of *L. sativum* are aperient, diuretic, tonic, demulcent, carminative, galactagogue, aphrodisiac, emmenagogue, poultice, and stimulant [21]. The seeds of this plant also possess rapid bone fracture healing ability [22]. They have been boiled with milk and are used to procure an abortion [23]. Fresh fruit is used for injuries, skin and eye disease. The leaves are antiscorbutic, diuretic, stimulant [21] and used for hepatic complaints [24]. Roots are bitter and acrid; it is useful in syphilis and tenesmus and used as a condiment [25]. According to the ethnobotanical studies conducted in different parts of Ethiopia, seeds of *L. sativum* are traditionally used for the treatment of malaria [26–31]. Crushed seeds are soaked in water, filtered and given orally in the morning for seven days for the treatment of malaria. *L. sativum* is known as Feto in Amharic *A. weloensis* is thought to be an effective medicinal remedy. It has been used for a long time in folk medicine for the treatment of constipation, burns, dermatitis, and wound [32]. Traditional healers of Ethiopia avowal the anti-malarial effect of *A.weloensis* in the Northern part of Ethiopia [33]. The leaf latex of *A. weloensis* is isolated and given orally to treat malaria. *A. weloensis* is known as Eret tafa in Amharic *L. gibberoa* is also thought to be an effective medicinal remedy. It has been used for the treatment of bronchitis asthma and chronic bronchitis. It has been also used topically for myositis and rheumatic nodules [34]. Traditional healers of Ethiopia claim the anti-malarial effect of *L. gibberoa* in different parts of Ethiopia [27, 30, 33]. Crushed roots are immersed in water, filtered and given orally for the treatment of malaria. *L. gibberoa* is known as Gibra in Amharic .This study was intended to evaluate the in vitro anti-malarial activity of these three plants since their in vitro anti-malarial activity has not been reported yet. In vitro tests of antimalarial have denoted some the most useful techniques for the study of the occurrence and changing pattern of drug resistance in the globe and have contributed essential evidence regarding a rationale management program based on the drug sensitivity patterns of the parasites. The resistance to antimalarials has stressed the necessity to identify and develop promising new antimalarials with different mechanisms of action to minimize the chance of cross-resistance with drugs which are in use nowadays. In vitro assay of drugs that are used to treat malaria have been widely used to support different studies associated to many phases of antimalarial drug investigation and development [35].

### Pharmacological activities of selected plants

A number of pharmacological activities of *L. sativum* have been reported in literatures. These include antibacterial [36–39], antifungal [40–42], anti-inflammatory [43], antioxidant and anti-cancer [44], hepatoprotective [45], antidiabetic [46, 47], analgesic [48], and antihypertensive [24] activities. *Aloe weloensis* has been investigated for antibacterial [49] and in vivo antimalarial [50] activities. *Lobelia gibberoa* has been evaluated for antimalarial pharmacological activity [51]. *Lobelia gibberoa* related species have been investigated for many pharmacological activities. These activities include anti-cancer [52], antioxidant and anti-inflammatory [53], and antiviral [54] activities of *Lobelia chinensis*, anti-inflammatory activity of *Lobelia laxiflora* [55], analgesic and antivenom activity of *Lobelia nicotianaefolia* [56], anti-inflammatory and anticonvulsant activity of *Lobelia flaccida* [57], and antimicrobial activity of *Lobelia pyramidalis* [58].

## Materials and methods

### Drugs, chemicals, and materials

The chemicals that were used in this study includes: dimethyl sulphoxide (DMSO) (LobaChemie.Pvt.Ltd, India), 5% hematocrit (human type A-positive erythrocytes, Italy), RPMI (Roswell Park Memorial Institute) 1640 medium (EuroClone, Celbio), 1% AlbuMax (Invitrogen, Milan, Italy), 0.01% hypoxanthine, 20 mM HEPES, 2 mM glutamine, absolute methanol (ALPHA CHEMIKA, India), the standard drug Chloroquine. In addition, the following instruments and materials: grinder (Hi-speed Multifunctional Grinder, Shanghai Yuan WO Industrial and Trade Co.Ltd.Yongkang City), maceration jar, orbital shaker (Stuart, UK), vacuum pump (PoongilCommercial.Co. Ltd, Yongsan-Gu.Seoul, Korea), muslin cloth, Whatman filters paper No.1, collecting flask, drying oven (GENLAB WIDNES, England), refrigerator,light microscope (OPTIKA, ITALY), frosted microscopic slides, measuring cylinder, 96-well flat-bottomed microplates, incubator,and spectrophotometer were used to perform the study.

### Plant materials collection and authentication

The seeds of *L. sativum* were purchased from Bahir Dar; the roots of *L. gibberoa* were collected from Delanta, and the leaf latex of *A. weloensis* from Woldia. The roots of *L. gibberoa* and the leaf latex of *A. weloensis* were collected after obtaining permission from the local administrative. Identification and authentication of the plants’ specimens was done by a taxonomist Getnet Chekole at the University of Gondar. After identification, voucher specimens of YW01/2011, YW02/2011, and YW03/2011 for *L .sativum*, *L. gibberoa* and *A. weloensis* respectively were deposited in Herbarium of University of Gondar for future reference. The roots of *L. gibberoa* and the leaf latex of *A. weloensis* were collected after getting permission from environmental protection office. Besides, the collected amount was very little that did not endanger the endemic plant species.

### Plant extraction

The seeds of *L. sativum* were purchased and cleaned; the roots of *L. gibberoa* were collected and washed with tap water to remove dirt and soil. After that, they were air-dried at room temperature under a shade and reduced to an appropriate size by grinding with a grinder machine. The powdered plant materials were extracted by cold maceration with 80% methanol for three consecutive days at room temperature. A total of 600 gm dried seeds of *L. sativum* (1:5, w/v) and 340 gm dried roots of *L. gibberoa* (1:8, w/v) were extracted in separate maceration jars for 72 h. Extraction was facilitated by using an orbital shaker at 120 rpm. The mixtures were first filtered using a muslin cloth and then with Whatman filter paper No. 1- which was assisted by a vacuum pump. The residues were re-macerated for another 72 h twice and filtered. The combined filtrates were dried in a drying oven at a temperature of 40^0^ C. The dried extracts were kept in a refrigerator at 4 °C until used [59]. The leaf latex of *A. weloensis* was collected by cutting the leaves transversally near the base and arranging them concentrically around a plate. The latex was then left in open shaded air for 3 days to allow evaporation of water [60].

### In vitro anti-plasmodial activity of extracts

The parasite lactate dehydrogenase (pLDH) assay was used to evaluate the in vitro anti-plasmodial activity of the crude extracts of the seeds of *L. sativum* and the roots of *L. gibberoa* as well as the leaf latex of *A. weloensis* against the CQ-sensitive (D10) and the CQ-resistant (W2) strains of *P. falciparum*. *Plasmodium falciparum* cultures were prepared according to the procedure described by Trager and Jensen, 1976 [61]. Both D10 and W2 strains were maintained at 5% hematocrit (human type A-positive erythrocytes) in RPMI 1640 medium supplemented with 1% AlbuMax, 0.01% hypoxanthine, 20 mM HEPES, and 2 mM glutamine. All the cultures were maintained at 37 °C in a standard gas mixture comprising 1% O_2_, 5% CO_2_, and 94% N_2_. Extracts were dissolved in DMSO (dimethyl sulphoxide) and then diluted with medium to get the required concentrations (final DMSO concentration was set to be < 1%, non-toxic to the parasite). Extracts were placed in 96-well flat-bottomed microplates and serial dilutions were made. Chloroquine was used as a standard drug. Synchronized cultures with a parasitemia of 1.5% and 1% final hematocrit were aliquoted into the plates and incubated for 72 h at 37 °C. Parasite growth inhibition was quantified spectrophotometrically by measuring the activity of the pLDH, according to a modified version of the method of Makler et al. [62] with slight modification [63]. The anti-plasmodial activity was expressed as 50% inhibitory concentrations (IC_50_); each IC_50_ value is the mean ± standard deviation of at least three separate experiments performed in duplicate.

### Data analysis

GraphPad Prism version 8 was used to compute the data obtained in this study and results were presented as Mean ± standard deviation (M ± SD). One-way analysis of variance (ANOVA) accompanied by Tukey’s honestly significance difference (HSD) *post-hoc* test was also carried out to compare mean of groups to each other. The analysis was performed with a 95% confidence interval and the significance was set at p < 0.05.

## Results

### Percentage yield

The percentage yield was determined for each plant using the method as follows:

Percentage yield = (Weight of extract/Weight of ground material) X 100.

After drying, a dry yellowish extract from *L. sativum* with 13.8% w/w yield and a gummy reddish extract from *L. gibberoa* with 14.4% w/w yield were obtained. A dark brown powder with 16.4% w/w yield was obtained from the leaf latex of *A. weloensis* which is the highest yield from all the three plants (Table 1).

**Table 1:** Percentage yield of crude extracts of *Lepidiumsativum*, *Lobelia gibberoa*, and *Aloe weloensis*

Extract	Actual mass(g)	Percentage yield(w/w)
***A.weloensis*** **latex**	194.5	16.4
*** L.gibberoa*** **extract**	49	14.4
*** L.sativum*** **extract**	82.8	13.8

### In vitro anti-plasmodial activity

**Fig. 1:**
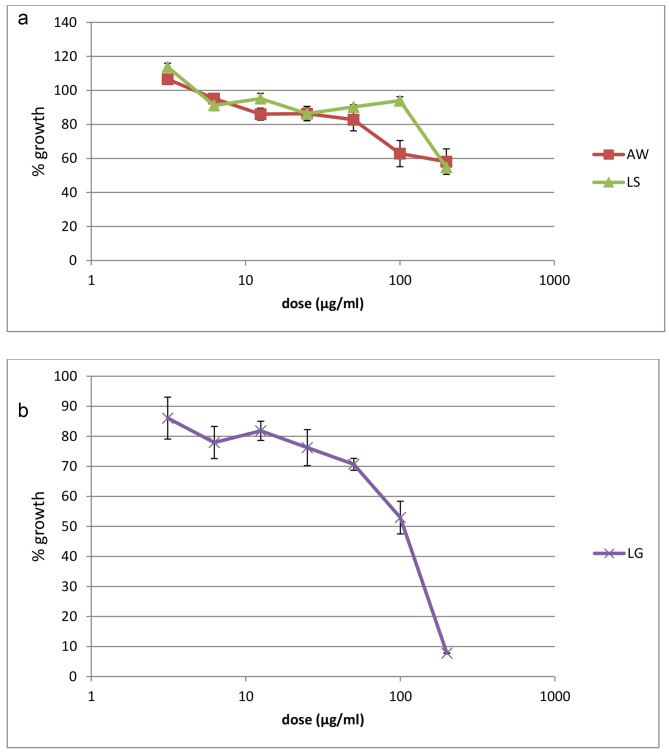
**a**: Plot of percentage growth of the CQ-sensitive *P. falciparum* D10 strain versus concentration of the plant extracts. The error bars indicates the SD of the triplicate results from a representative experiment out of three; AW, leaf latex of*A. weloensis*; LS, methanolic extract of *L.sativum* seeds. **b**: Plot of percentage growth of the CQ-sensitive *P. falciparum* D10 strain versus concentration of the plant extracts. The error bars indicates the SD of the triplicate results from a representative experiment out of three; LG, methanolic extract of *L.gibberoa* roots

**Fig. 2:**
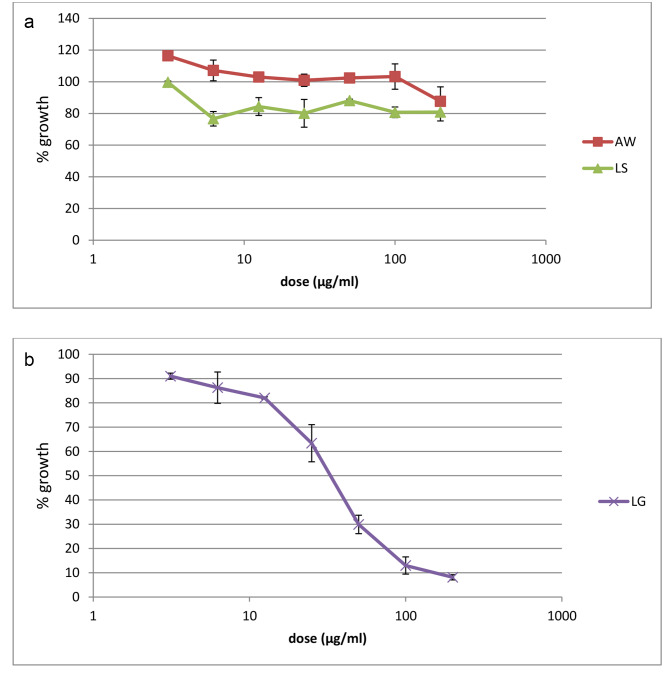
**a**: Plot of percentage growth of the CQ-sensitive *P. falciparum* W2 strain versus concentration of the plant extracts. The error bars indicates the SD of the triplicate results from a representative experiment out of three; AW, leaf latex of *A. weloensis*; LS, methanolic extract of *L .sativum* seeds. **b**: Plot of percentage growth of the CQ-sensitive *P. falciparum* W2 strain versus concentration of the plant extracts. The error bars indicates the SD of the triplicate results from a representative experiment out of three; LG, methanolic extract of *L. gibberoa* roots

The anti-plasmodial activity of the tested medicinal plants’ extracts against the CQ-sensitive D10 and CQ-resistant W2 strains of *P. falciparum* is presented in Fig. 1**and** Fig. 2, respectively. The methanolic extract of *L.gibberoa* roots caused a concentration-dependent growth inhibition against both strains of *P. falciparum*. Among the three extracts, the methanolic extract of *L. gibberoa* roots showed the highest in vitro anti-plasmodial effect against both D10 and W2 *P. falciparum* strains. The half-inhibitory concentration (IC_50_ values) of the selected plants extracts is presented in Table 2. The methanolic extract of *L. gibberoa* roots showed the lowest IC_50_ value when compared with the other extracts against both the CQ-sensitive *P. falciparum* D10 strain (IC_50_ = 103.83 ± 26.17 µg/mL) and CQ-resistant *P.falciparum* W2 strain (IC_50_ = 47.11 ± 12.46 µg/mL). However, the methanolic extract of *L. sativum* seeds and the leaf latex of *A. weloensis* were not active with an IC_50_ value > 200 µg/mL against both D10 and W2 strains (Table 2).

**Table 2:** In vitro anti-plasmodial activity against *Plasmodium falciparum* strains, D10 (CQ-sensitive) and W2 (CQ-resistant)

Extracts	D10 IC_50_(µg/mL)		W2 IC_50_(µg/mL)	
	Mean	SD	Mean	SD
**AW**	> 200		> 200	
**LG**	103.83	26.17	47.11	12.46
**LS**	> 200		> 200	
**CQ (ng/mL)**	11.25	1.96	150.14	18.1

Note: Data are expressed as mean ± SD of three different experiments, each performed in duplicate; AW, leaf latex of *A. weloensis*; LG, methanolic extract of *L.gibberoa* roots; LS, methanolic extract of *L sativum* seeds; CQ, chloroquine

## Discussion

In the present study, the in vitro anti-plasmodial activity of the methanolic extracts of *L. gibberoa* roots and *L. sativum* seeds, as well as the leaf latex of *A. weloensis* were investigated. In discovering new anti-malarial drugs, history has taught us how the knowledge of traditional medicines is valuable [12–14]. The in vitro anti-plasmodial effect of three medicinal plants’ extracts was screened against a CQ-sensitive D10 and CQ-resistant W2 strains.

Among the three selected plants used for the traditional treatment of malaria in Ethiopia, the methanolic extract of *L. gibberoa* roots exhibited a moderate in vitro anti-plasmodial activity against asexual forms of *P. falciparum* whereas the methanolic extract of *L. sativum* seeds and the leaf latex of *A.weloensis* were inactive. The methanolic extract of *L. gibberoa* roots showed the lowest IC_50_ value against both strains of *P. falciparum* used. In one study, the in vivo antimalarial activity of the 80% methanolic extract of *L. gibberoa* (400 mg/kg), methanol fraction (400 mg/kg), and lobetyolin isolate (100 mg/kg) was evaluated and they exhibited antimalarial activity with chemosuppression values of 73.05, 64.37, and 68.21%, respectively [51]. This result supports the in vitro antimalarial activity and the traditional use of the plant for the treatment of malaria.

Various studies on the antimalarial activity of the genus *Aloe* have been reported. In one study, ether leaves extracts of *A. dawei* showed a potential inhibition of parasite growth against *P. falciparum* with the IC_50_ value of 7.965 µg/mL [64]. Similarly, *Aloe perryi* has been studied for its in vitro anti-plasmodial activity and the result supports the use of the plant leaves latex in the treatment of malaria with the IC_50_ value of 60.60 µg/mL [65]. In vitro anti-plasmodial activity test of aqueous leaf extract and isolated compounds of *A .vera* collected from different climatic regions in India has been conducted. Different chemosuppressive effect of samples collected from different climatic area has been observed with EC_50_ values ranging from 0.289 to 1056 μg/mL [66]. Similarly, the anti-malarial activity of the leaf latex of *A. citrina* and the leaf latex of *A. megalacantha* showed a significant dose-dependent chemo-suppression activity against *P. berghei* in Swiss albino mice with the highest parasitemia suppression of 60.59% [67] and 79.6% [68], respectively at the higher dose tested (400 mg/kg). Other studies on the leaf latex of *A. percrassa* and *A. macrocarp* showed a significant dose-dependent chemo-suppression activity against *P. berghei* in Swiss albino mice with the highest parasitemia suppression of 73.6% [60] and 74.3% [69], respectively at the higher dose tested (400 mg/kg). In one study, the in vivo antimalarial activity of the leaf latex of *A. weloensis* was evaluated and results showed that the plant has a significant dose-dependent chemo-suppression activity against *P. berghei* in Swiss albino mice with the highest parasitemia suppression of 66.4% [50]. Conversely, in the present study, the leaf latex of *A. weloensis* was inactive (IC_50_>200 µg/mL). This might be that the leaf latex of *A. weloensis* might have inactive molecules that need biological transformation (metabolism) into active molecules. These types of drugs are known as reactionary drugs or pro-drugs.

There are limited studies on the anti-malarial activity of *Lepidium* genus. In one study, the in vitro anti-malarial activity of *Lepidium virginicum* has been conducted and results showed that this extract was inactive [70]. Similarly, in the present study, the methanolic extract of *L. sativum* seeds was inactive (IC_50_ > 200 µg/mL). The methanolic extract of *L. sativum* seeds *might* have inactive molecules that need biological transformation (metabolism) into active molecules. Thus, studying the in vivo anti-malarial property of this plant extract is necessary.

Phytochemicals or secondary metabolites (anthraquinones, flavonoids, phenols, terpenoids, tannins, glycosides and others) showed antimalarial activity in different plants extracts through various mechanism of action [71–74]. Moreover, flavonoids which have antioxidant activity may also contribute to the antimalarial activity. Antioxidant compounds can inhibit hemozoin formation, and free heme is very toxic for malaria parasite [75]. In addition, secondary metabolites such as glycosides have been shown to possess direct antiplasmodial effects [76]. Therefore, the antimalarial activity of plants could be elicited from single or synergetic action of these metabolites.

## Conclusion

The methanolic extract of *Lobelia gibberoa* roots showed a promising in vitro anti-plasmodial activity against *P. falciparum*. Thus, this study partly justifies and may also support the traditional use against malaria. However, the methanolic extract of *L sativum* seeds and the leaf latex of *A. weloensis* did not exert in vitro suppressive activity on the growth of *P. falciparum* strains. The methanolic extract of *Lepidium sativum* seeds may also be examined for its in vivo anti-malarial activity to determine if there is/are reactionary drug/s that needs biological transformation to act.

## Data Availability

The data used and analyzed in this study are available from the corresponding author on reasonable request.

## Abbreviations and Acronyms

ACT: Artemisinin-based Combination Therapy
ANOVA: Analysis of Variance
CDC: Centers for Disease Control and Prevention
CQ: Chloroquine
DMSO: Dimethyl Sulfoxide
HSD: Honestly Significance Difference
IC_50_: Half inhibitory amount of a substance
pLDH: Parasite Lactate dehydrogenase
RPMI: Roswell Park Memorial Institute
WHO: World Health Organization

## Declarations

### Plant collection and Authentication

Identification and authentication of the plants’ specimens was done by a taxonomist Getnet Chekole at the University of Gondar and it was in line with characteristics that is published in the monograph.

### Method of plant collection

All methods of plant collection complied with a guideline developed in America by the Plant Conservation Roundtable,Washington,D.C., Adopted June 18, 1986. This guideline is known as BOTANY 440/540.
